# Autogenous vaginal tunic used as reinforcement in inguinal herniorrhaphy in a dog

**DOI:** 10.29374/2527-2179.bjvm008225

**Published:** 2026-03-12

**Authors:** Milene Costa da Silva, Marcondes Pessoa de Freitas, Beatriz Barreto Gomes, Victória Ranna Dias dos Santos, Ester Morais Alves Pereira Franca, Eduardo Melo Nascimento, Karoline Pereira Silva Rodrigues, João Paulo Vitória do Nascimento, Deusdete Conceição Gomes

**Affiliations:** 1 Hospital Veterinário Universitário, Universidade Federal do Oeste da Bahia, Barra, BA, Brazil

**Keywords:** hernia, dogs, surgery, biological graft, hérnia, cão, cirurgia, enxerto biológico

## Abstract

This case report describes the use of an autologous vaginal tunic as a reinforcement material in the repair of an inguinal hernia in a 10.5-year-old intact male mixed-breed dog, presented to the Veterinary Hospital of the Federal University of Western Bahia with a 10-month history of swelling in the right lateral region of the penis. The patient was diagnosed with prostatic hyperplasia associated with an inguinal hernia. Surgical treatment consisted of inguinal herniorrhaphy; given the muscle weakness observed intraoperatively, the inguinal wall was reinforced using a double-layered autologous vaginal tunic obtained following bilateral orchiectomy. The procedure resulted in successful recovery, with no postoperative complications, demonstrating tissue stability and excellent anatomical integration. One year and four months later, the animal was euthanized due to complications arising from multicentric lymphoma. Necropsy revealed firm integration between the vaginal tunic and adjacent tissues, with the abdominal cavity preserved in its entirety. Histopathological analysis confirmed the preservation of muscle, adipose, and connective tissues, along with the absence of inflammation, granulation tissue, hyperplasia, or fibrosis. As this is a single case report the present findings do not allow for generalizations. However, they suggest that the autologous vaginal tunic may represent a viable, safe, and cost-effective alternative as a reinforcement material in canine inguinal herniorrhaphy.

## Resumo

Objetiva-se, com este relato, detalhar o uso da túnica vaginal autógena como material de reforço no reparo de uma hérnia inguinal em um cão macho, sem raça definida, de 10 anos e 6 meses, atendido no Hospital Veterinário da Universidade Federal do Oeste da Bahia, com queixa de aumento de volume na região lateral direita do pênis, com evolução de 10 meses, diagnosticado com hiperplasia prostática associada à hérnia inguinal. O tratamento consistiu em herniorrafia inguinal, durante a qual foi identificada fragilidade muscular, optando-se pelo reforço da parede com a utilização da túnica vaginal autógena, obtida após orquiectomia bilateral. O procedimento resultou em recuperação cirúrgica bem-sucedida, sem complicações pós-operatórias, com estabilidade tecidual e excelente integração anatômica. Após um ano e quatro meses, o animal foi eutanasiado devido a complicações de linfoma multicêntrico, em que, na necropsia, constatou-se fixação entre a túnica vaginal e os tecidos adjacentes, com preservação da integridade da cavidade abdominal. A histopatologia revelou preservação dos tecidos muscular, adiposo e conjuntivo, sem sinais de inflamação, tecido de granulação, hiperplasia ou fibrose. Por se tratar de um relato de caso único, os achados aqui descritos não permitem generalizações. Contudo, sugerem que a túnica vaginal autógena pode constituir uma alternativa viável, segura e de baixo custo como material de reforço na herniorrafia inguinal em cães.

## Introduction

Inguinal hernia occurs when abdominal contents protrude through the inguinal ring, a structure defined by the region's muscular fascia (Oliveira et al., 2023). Common herniated contents include the omentum, intestines, prostate, bladder, uterus, and colon (Borges et al., 2022). Clinical signs vary depending on the specific herniated content and the duration of the condition (Fossum, 2019). Typically, a soft swelling is observed in the inguinal region upon palpation, although a firm, painful mass with local hyperthermia may suggest incarceration or strangulation of the herniated (Borges et al., 2022).

Diagnosis is primarily based on the case history, palpation of the swelling, inspection of the inguinal ring diameter, evaluation of the characteristics and reducibility of the herniated content, and the presence of adhesions (Oliveira & Silva, 2021). Imaging techniques are often recommended to help identify and assess the viability of herniated organs. The treatment of choice for inguinal hernia correction is surgical herniorrhaphy, which involves reduction and repositioning of the contents and narrowing of the external inguinal ring (Checchinato et al., 2024). When direct tissue approximation is unfeasible due to tissue loss or excessive tension at the suture line, biological or synthetic materials can be used to reinforce the repair, thus minimizing the risk of complications such as dehiscence, eventration, or evisceration (Guerios et al., 2020).

The vaginal tunic is a serous membrane that envelops the spermatic cord and testicle. It has previously been utilized as an autologous graft for diaphragmatic repair (Tanaka et al., 2004) and pelvic diaphragm reconstruction in dogs with perineal hernia (Faria et al., 2020), with satisfactory outcomes. Therefore, the objective of the present report is to describe the use of a free, double-layered autologous vaginal tunic as reinforcement for inguinal herniorrhaphy in a dog, as well as to evaluate its integration with surrounding tissues.

## Case report

A 10-year-and-six-month-old, 24.3 kg, intact male mixed-breed dog was presented to the Veterinary Hospital. During anamnesis, the owner reported a primary complaint of swelling on the right lateral region of the penis, with had an approximate duration of ten months. The owner also stated that the animal had been previously treated by a clerk at an agricultural supply store, with no clinical improvement. On physical examination, the dog was alert, with no apparent dehydration, non-reactive lymph nodes, pink mucous membranes, capillary refill time less than two seconds, body temperature of 38.8 ºC, heart rate of 140 bpm, tachypnea (60 breaths per minute), and no ectoparasites. The patient had a good body condition score. During palpation of the right inguinal region, a soft-consistency mass was identified; it was painless, non-inflammation, and irreducible.

Given the suspicion of an irreducible inguinal hernia, a complete blood count and biochemical profile (alanine aminotransferase [ALT], urea, creatinine, albumin, alkaline phosphatase [ALP], and globulins), as well as abdominal ultrasonography focusing on the inguinal swelling, were requested. Laboratory tests were within normal limits for the patient's species and age. Ultrasonographic findings revealed an enlarged prostate with an asymmetric shape, irregular contours, heterogeneous echotexture, and cystic structures within the parenchyma. Scanning of the subcutaneous mass in the right inguinal region revealed discontinuity of the abdominal musculature, suggesting a hernial ring, with hyperechoic tissue overlying an anechoic content, however, definitive identification of herniated content was not possible.

Based on the examinations performed, the diagnosis of inguinal hernia was confirmed, and inguinal herniorrhaphy was indicated. During the patient’s preoperative evaluation, physiological parameters were within normal limits for the species, and the patient was classified as ASA II based on the anesthetic risk assessment. Following an 8-hour fast from solid food and a 4-hour water fast, the patient was taken to the preoperative room. A wide trichotomy was performed on the ventral abdominal region, the medial thigh, and the lumbosacral area. Vascular access was established for fluid therapy with lactated Ringer’s solution (1 drop per second). Pre-anesthetic medication administered intramuscularly consisted of chlorpromazine^3^ (1.1 mg/kg) combined with morphine^3^ (0.3 mg/kg).

Meloxicam^2^ (0.2%; 0.2 mg/kg) was administered subcutaneously; sodium dipyrone (500 mg/mL; 25 mg/kg) was administered intravenously; and enrofloxacin^1^ (5 mg/kg, IM) was also given. Approximately five minutes after the administration of the pre-anesthetic medication, with visible muscle relaxation, the patient was transferred to the surgical suite. Anesthetic induction was performed using ketamine (1 mg/kg, IV) followed by propofol^3^ (3 mg/kg, IV). After induction, the patient was positioned in sternal recumbency and orotracheal intubation was performed with a size 8.0 Magill tube. The surgical anesthetic plan was maintained with inhalation anesthesia using sevoflurane delivered via a universal vaporizer, in a semi-closed system, with an oxygen flow rate of 1.5 L/min. Epidural blockade was subsequently performed using 0.5% bupivacaine^8^ (2 mg/kg via the epidural route).

The patient was positioned in dorsal recumbency with the aid of a trough table, and urethral catheterization was performed using a size 10 catheter. Surgical field antisepsis was carried out using an alcoholic solution of chlorhexidine digluconate^7^ (0.2%). After securing the surgical drapes with Backhaus towel clamps, the procedure was initiated with an oblique skin incision along the major axis of the swelling in the right inguinal region, approximately 10 cm in length, cranial to the pelvic brim. The incision was deepened through the subcutaneous tissue until the hernial sac was visualized, followed by blunt digital dissection to expose the sac. Upon opening, the hernial contents were identified as omentum and intestinal loops. The herniated contents were reduced into the abdominal cavity, and subsequent palpation of the contralateral internal ring revealed no abnormalities. The hernial sac was transected at its base and closed using a continuous simple suture pattern with absorbable polyglactin 910 thread (2-0). After closing the hernial sac, the diameter of the hernial ring was reduced using interrupted simple sutures with non-absorbable monofilament nylon thread (2-0), leaving sufficient space for the passage of a hemostatic forceps to prevent damage to the external pudendal artery and vein and the genitofemoral nerve.

During the muscle suturing, excessive tension and muscle fragility were identified, which raised concern for suture dehiscence in the immediate postoperative period. Therefore, reinforcement of the herniorrhaphy was performed using an autologous vaginal tunic obtained following bilateral orchiectomy.

To obtain the vaginal tunic, a longitudinal incision was made in the pre-scrotal region, in a craniocaudal direction, through the skin and subcutaneous tissue. Upon exteriorizing the testicle, orchiectomy was performed using the closed technique, with ligation of the spermatic cord using 0 nylon thread. No bleeding was observed, and the same technique was applied to the contralateral testicle through the same incision. The subcutaneous tissue was approximated using an intradermal suture pattern with absorbable polyglactin 2-0 thread, and the skin was closed with simple interrupted sutures using 2-0 nylon thread.

**Figure 1:**
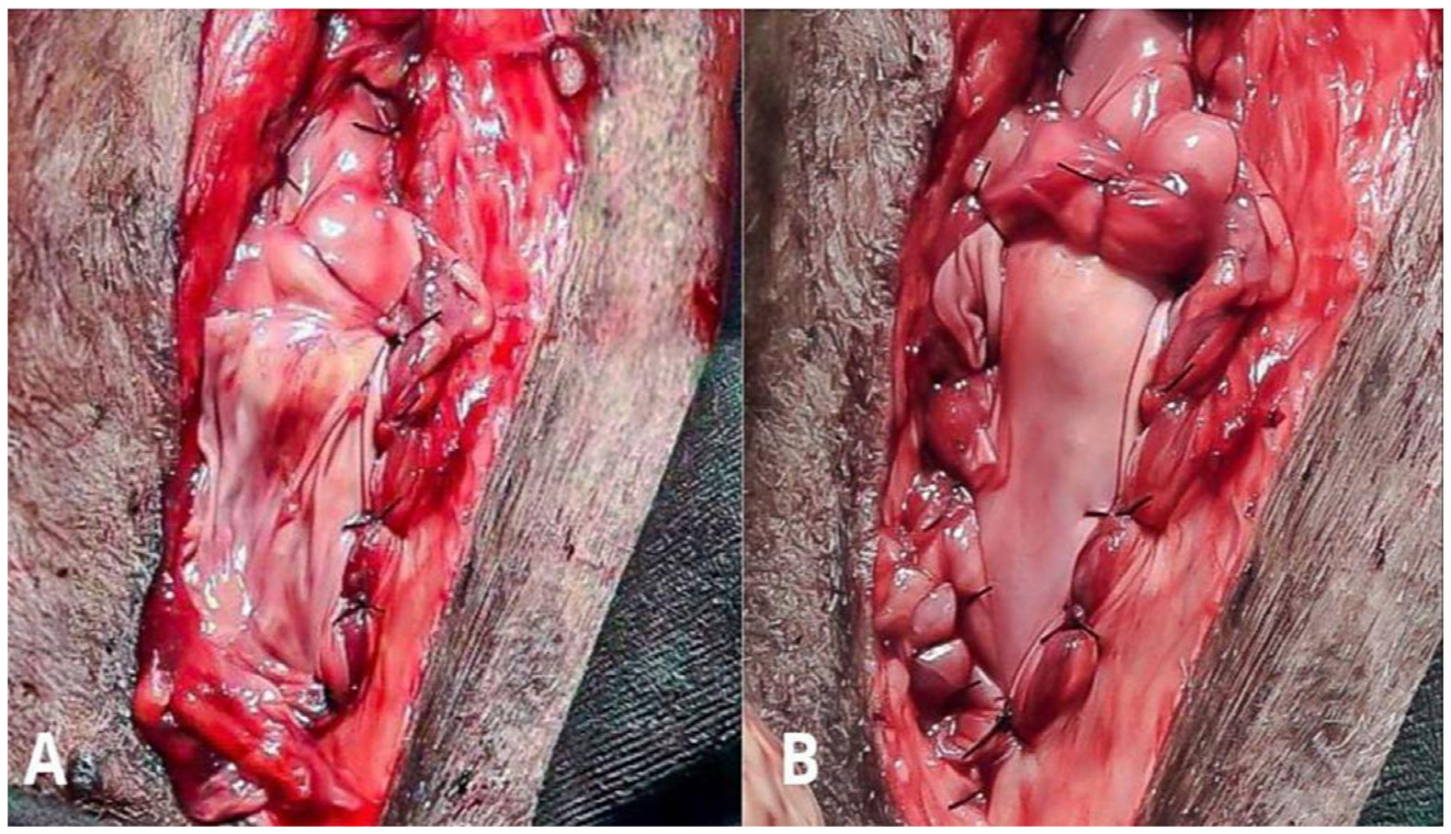
Intraoperative image showing the fixation of the graft.

After orchiectomy, the vaginal tunic was opened, forming a flat membrane, and was fixed to the inguinal musculature in a double layer, using simple interrupted sutures at the edges and center of the graft with 0 nylon thread (Figure 1).

The subcutaneous tissue was closed using an intradermal suture pattern with absorbable synthetic polyglactin 910 thread 2-0, including muscular anchoring sutures to reduce the dead space created by tissue dissection. Skin closure was performed using a Wolff suture pattern with 2-0 nylon thread. At the end of the surgical procedure, the wound was cleaned with physiological saline solution, and a healing spray containing silver sulfadiazine and neomycin sulfate was applied. The surgical area was covered with sterile gauze and microporous tape.

In the immediate postoperative period, tramadol hydrochloride^3^ (2 mg/kg subcutaneously) was administered. Cephalexin 500 mg (30 mg/kg, orally, twice daily) was prescribed for 10 days, meloxicam 2.0 mg (0.1 mg/kg orally, once daily) for 3 days, and tramadol hydrochloride 100 mg/mL (3 mg/kg; 12 drops orally, three times daily) for 5 days. Postoperative care included daily cleaning of the surgical wound with physiological saline solution (0.9% NaCl) and application of rifamycin spray (10 mg/mL, once daily) for 10 days, along with the use of an Elizabethan collar until suture removal.

The patient returned for postoperative evaluations five days after the procedure, during which general condition, presence or absence of seroma at the surgical wounds (herniorrhaphy and orchiectomy), suture dehiscence, and local infection were assessed. The patient was found to be alert with no signs of seroma or suture dehiscence. After 10 days, a reduction in prostatic volume was observed, with no signs of recurrence or possible complications. The skin sutures were removed, and the patient was discharged from postoperative follow-up.

**Figure 2:**
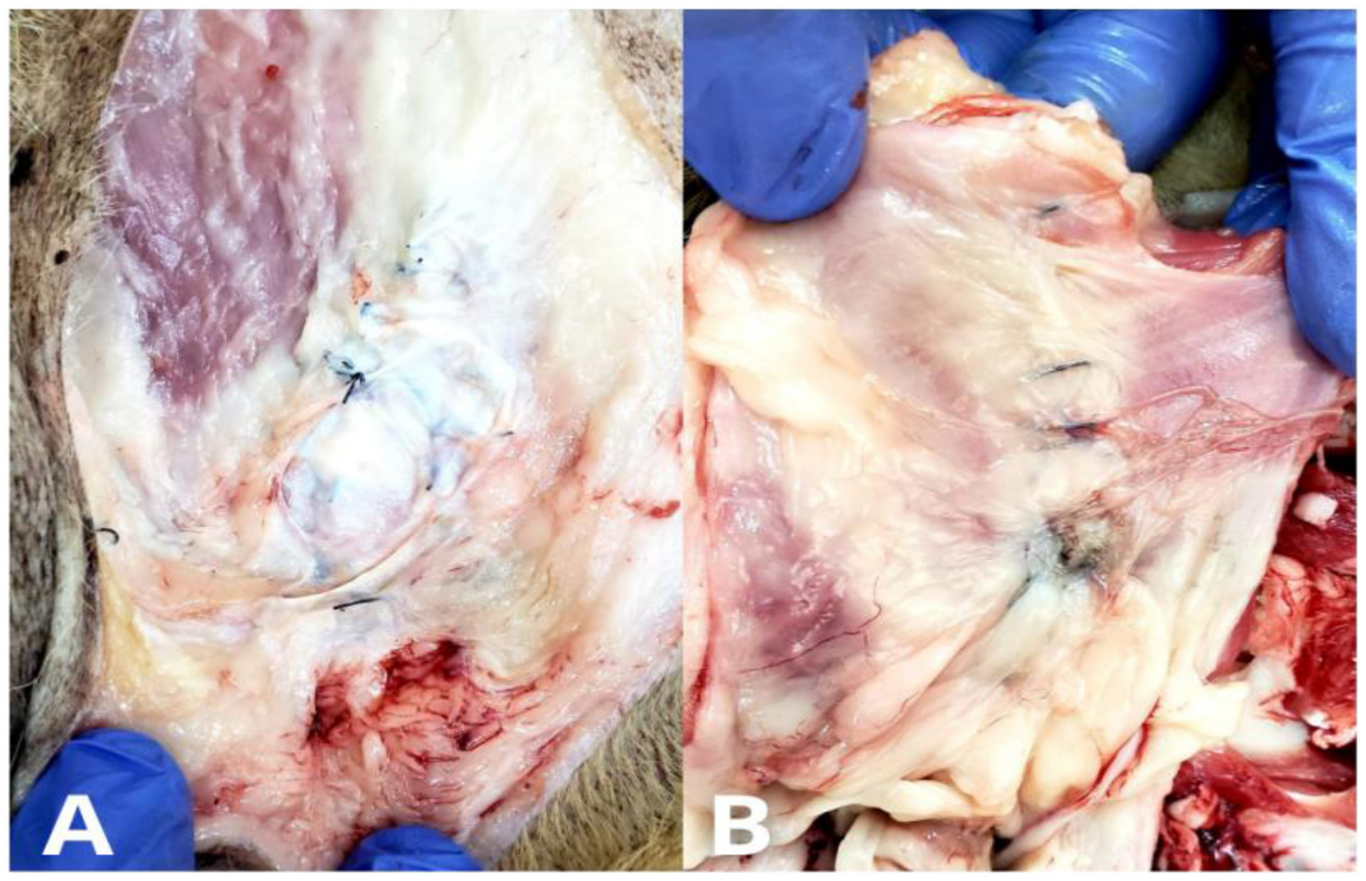
Image obtained during the animal’s necropsy showing adipose tissue covering the external surface (A) and peritoneum covering the internal surface (B).

Ten months later, the patient returned to the veterinary hospital and was diagnosed with multicentric lymphoma. Six months after diagnosis, due to lymphoma-related complications, the patient was euthanized. Subsequently, a necropsy was performed, which included inspection of the graft implantation site. Adipose tissue covering the surgical area and some nylon sutures were identified. Upon accessing the abdominal cavity, dissection of the peritoneum and musculature was carried out until the union between the muscle tissue and the vaginal tunic was identified, with preserved integrity of the external region. Upon removal of the fragment, perfect stability and healing were confirmed, maintaining elasticity and isolation of the cavity. On palpation, integrity and union of the muscle tissue to the vaginal tunic graft were noted. No sacculations were observed, and after dissection, the herniorrhaphy sutures and graft fixation were maintained (Figure 2).

**Figure 3:**
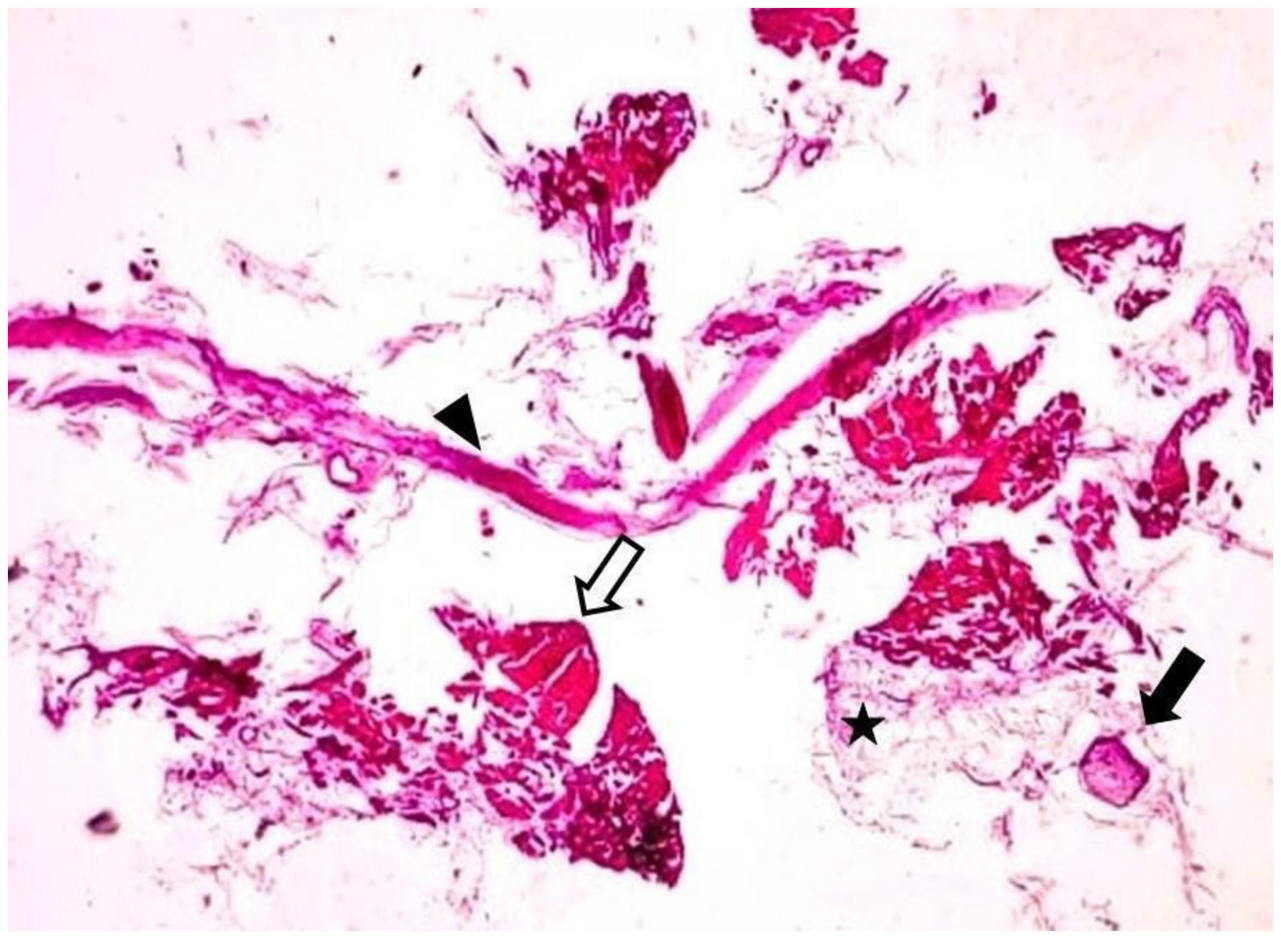
Integration of the vaginal tunic with the animal’s tissue. The vaginal tunic (arrowhead) is located between adipose tissue (star), muscular and connective tissues (white arrow), and peripheral nerve (black arrow). Hematoxylin and eosin stain; scale bar = 100 μm. Objective lens 10x.

Following macroscopic analysis, a fragment of the graft was collected and fixed in 10% formalin. Then, the sample was sent to the Pathology Department of Veterinary Medicine. For histological slide preparation, the sample was embedded in paraffin, sectioned into 5–6 µm slices, stained with Hematoxylin and Eosin (H&E) following Junqueira & Carneiro’s protocol (2018) for further histological analysis. Histologically, the organization of adipose, and muscular, and connective tissues was observed, including the presence of blood vessels and peripheral nerves, demonstrating physiological stability and integrity among the tissues. The vaginal tunic was identified by elongated collagen fibers arranged in parallel and longitudinal orientation, with elongated nuclei and venules present. Additionally, a peripheral ganglion was observed within the grafted tissue, with no evidence of granulation tissue, hyperplasia, or fibrosis (Figure 3).

## Discussion

The age of the animal in the present study is a noteworthy factor, as it aligns with the most common age range for the occurrence of inguinal hernia in the canine species. This finding is supported by the literature, which frequently associates aging with an increased incidence of certain clinical conditions, including inguinal hernias. According to Vasconcelos et al. (2020), dogs older than four years show a higher predisposition to pathological changes like those observed in the current case. The reported study involves an intact male dog, a situation rarely documented in the literature (Borges et al., 2022), which may be related to the underreporting of the condition in males.

In the present case, the exact cause of the inguinal hernia was not fully elucidated. The most relevant finding was the fragility of the inguinal ring in a large-breed dog weighing 24.3 kg. The observed fragility of the inguinal ring could be associated with increased intra-abdominal pressure caused by the patient’s body weight, supporting Philip et al. (2019), who suggests that fat accumulation may cause dilation of the vaginal process and inguinal ring, thereby facilitating hernia formation. Crucially, in this report, the hernia showed signs of incarceration, characterized by irreducibility on palpation and reduction only being possible during surgery following adhesion dissolution.

It is important to highlight that implants, typically composed of polypropylene mesh, are commonly employed when direct tissue approximation is not feasible due to the defect size, particularly in inguinal hernias. Therefore, synthetic mesh could have been applied in this case, given its inert nature and mechanical strength. In contrast, biological grafts represent an effective and cost-efficient alternative for the repair of tissue defects, especially in contexts where synthetic materials are unavailable or when financial constraints limit owner access to them. In this regard, the preference for utilizing an autologous biological graft was driven not only by the need to reinforce the repaired area but also by potential for rapid tissue regeneration, reduced risk of immunological reactions, and absence of additional procedural costs.

The utilization of the vaginal tunic has been reported in various surgical procedures due to its advantageous properties, such as its ready availability in intact male dogs, good integration with recipient tissue, and a minimized inflammatory response. Among its applications, correction of pelvic diaphragm defects (Faria et al., 2016; Saigali et al., 2017; Guerios et al., 2020) and lamellar keratoplasty in dogs (Vicenti et al., 2002) have been described, as well as its use as a biological dressing for the treatment of cutaneous wounds in rats (Campos et al., 2021). The use of the vaginal tunic, whether autologous or heterologous, is widely valued due to its availability and favorable biological characteristics, including good tissue integration and minimal inflammatory reaction. Despite these promising outcomes reported in the literature regarding the application of the vaginal tunic in diverse contexts, its specific use as a reinforcement for inguinal herniorrhaphy had not yet been documented. This gap in the literature, coupled with the reported advantages and its ready availability since the patient was intact justified its selection as an effective and innovative alternative for the repair of inguinal defects, providing the necessary support for neotissue formation and reinforcement of the inguinal muscle repair.

Another important point to highlight is the method used for harvesting the vaginal tunic, which proved to be simpler than that described by other authors, such as Borges et al. (2022), who opted for inguinal hernia repair following a scrotal orchiectomy using a mesorchium flap. In that report, the procedure required a delicate dissection to obtain the flap, which differs from the technique employed in the present study, which involved a closed pre-scrotal orchiectomy, as described by Fossum (2019). The closed technique allowed for better preservation of the vaginal tunic, maintaining the structural integrity of the tissue during the orchiectomy. In the case presented, a free graft transposition technique was chosen due to its simplicity, allowing the vaginal tunic to be applied to any region of the body. This method essentially involves harvesting the membrane after orchiectomy (Guerios et al., 2020), followed by its placement and fixation in the recipient site, which requiring less surgical skill and reduces operative time.

Philip et al. (2019) discussed inguinal herniorrhaphy in dogs, noting that in some cases, polypropylene mesh was used for reinforcement. The study emphasized the need for surgical drains to minimize seroma formation in patients with dead space. However, in the present case, no drains were used. The dead space resulting from subcutaneous tissue dissection was minimized through the use of intradermal sutures and muscular anchoring stitches. Follow-up until suture removal, which occurred 10 days postoperatively, revealed proper wound healing without significant signs of edema, pain, or erythema. Crucially, there was no seroma accumulation, local infection, or suture dehiscence, thereby demonstrating the effectiveness of the technique used.

The integrity of the muscular and adipose tissues following graft placement indicates that the healing process was efficient and that the graft was well tolerated by the host organism (Campos et al., 2021). In a study using the vaginal tunic preserved in 98% glycerin as a biological dressing for the treatment of cutaneous wounds in Wistar rats, the same author reported the presence of inflammatory infiltrate, fibrin deposition, and connective tissue formation, along with re-epithelialization. However, unlike the aforementioned study, an initial evaluation of the tissue response was not possible in the present case. The results of the histological assessment revealed the integrity of the muscular, adipose, and connective tissues, in addition to the absence of granulation tissue, hyperplasia, or fibrosis. These findings suggest that the healing process at the graft site occurred successfully, without apparent complications, such as infection or rejection (Canellas et al., 2017).

Given the above and all the characteristics highlighted throughout this study demonstrating satisfactory outcomes in terms of stability, integrity, and tissue healing, it is also important to emphasize that the preservation and integrity of a peripheral ganglion further supports the conclusion that the implanted tissue underwent successful regeneration. This is particularly relevant considering that nervous tissue cells are among the first to degenerate in cases of tissue injury or regenerative failure (Junqueira & Carneiro, 2018). Hence, the presence of nervous tissue represents an additional finding that reinforces the effectiveness of the technique employed in the present study.

## Conclusions

The approach adopted in this study, using an autologous vaginal tunic proved to be an effective and viable alternative for reinforcing inguinal herniorrhaphy in the dog. The technique resulted in satisfactory outcomes in terms of tissue stability and tissue healing, with no signs of recurrence or complications during the follow-up period. Furthermore, this method offers new possibilities for the treating inguinal hernias in dogs, especially in cases where synthetic implants are unavailable or when financial constraints limit the owner’s options.
